# Review of: Appalachians for Medicaid Website

**DOI:** 10.13023/jah.0303.10

**Published:** 2021-07-25

**Authors:** Kendra Barker

**Affiliations:** West Virginia University

**Keywords:** Appalachia, review, website, media

## Abstract

The *Journal of Appalachian Health* is committed to reviewing published media that relates to contemporary concepts affecting the health of Appalachia. Access to care and the health disparities we face have a direct effect on our experience of illness. Dr. Kendra Barker reviews the website: *Appalachians for Medicaid*.

## MEDIA TYPE: WEBSITE

### CITATION

*Sharing our Neighbor’s stories*. Appalachians for Medicaid. (2021, January 20). https://appalachiansformedicaid.org/.

Cost: This is a free-access, public website with nothing behind a pay wall.

### ABOUT THE REVIEWER

Kendra Barker is an Associate Professor at the West Virginia University School of Nursing. She has 15 years of experience as an advanced practice registered nurse in rural West Virginia. She is a Certified Nurse Educator, Certified Diabetes Educator, and Board-Certified Family Nurse Practitioner. She sits as a member of the Board for the Department of Public Health in Preston County, West Virginia. As a Faculty member and Doctorate of Nursing Practice, she has developed her expertise in primary care for chronic diseases including diabetes and substance use disorder management. Her publications relate to these subjects and telehealth.

### ABOUT THE WEBSITE

The website is from the prospective of health justice advocates in Appalachia and is the result of a combined effort from Kentucky Voices for Health, Tennessee Health Care Campaign, UHCAN Ohio, West Virginians for Affordable Health Care, and Supporting Partner Community Catalyst. This site shares both statistics about Appalachian healthcare as well as stories told by individuals in Appalachia, with particular attention to issues of healthcare access and Medicaid healthcare coverage. There are stories shared by individuals receiving healthcare as well as healthcare providers.

### THE REVIEW

The Appalachians for Medicaid website implores both society and legislators to realize the need that exists for Medicaid healthcare coverage to be available to all individuals living in Appalachia. As a health care provider, the information shared on this site resonates with me and correlates to my personal experiences with individuals needing Medicaid coverage. Many individuals struggle in the “middle class,” with too much income to qualify for government programs or assistance, but not enough to pay for healthcare needs. Multiple statistics about Medicaid are shared on this website, which also describes an alliance between healthcare advocates from four states in Appalachia (Kentucky, Tennessee, Ohio, and West Virginia). Advocates are working to collect stories and spread awareness of healthcare issues related to Medicaid coverage, including access to care problems, financial issues, and the vast degree of health outcomes affected by having Medicaid coverage. The alliance is supported by the “Community Catalyst” consumer advocacy group, with a shared belief that affordable, quality health care should be accessible to everyone. Any individuals, community interest groups or legislators considering important decisions about the health system related to Medicaid coverage for Appalachians should visit this site to learn firsthand about these issues from the stories shared in both written and video formats. At the time of this review, 12 compelling individual stories are shared with experiences across the lifespan and the states of Appalachia describing Medicaid benefits.

While Medicaid is the federal government public health insurance program, flexibility is given to states in how to administer the program within federal guidelines. According to the site, 1 in every 3 to 5 people in these Appalachian states rely on Medicaid coverage. Since states have individual discretion with the qualifying criteria, there are discrepancies between states. The site lists statistics but is lacking references and identification of a sponsor. Some terms and statistics do have links referring to corresponding websites. According to this website, Tennessee hasn’t adopted the state guidelines and provided access like the other states. I was left with the impression that one purpose of this site is to provide leverage for Tennesseeans to convince lawmakers in Tennessee to expand access to Medicaid. Individuals in Tennessee are noted to be “experiencing worsening racial health disparities, rural hospital closures, and … unable to access the health care they need.”

One might think that in the growing age of telemedicine and the COVID 19 pandemic, expanded healthcare coverage and access are available. However, access to telemedicine services may be compromised with technology and communication challenges, including poor internet access in rural areas, and Medicaid coverage is still needed to pay for these services. As an Appalachian, I struggle with poor internet access on a regular basis, so I am aware of the challenge that this presents to deliver adequate healthcare via telehealth. While it is a promising option for the future, it is not the ultimate answer to current healthcare dilemmas.

By sharing the stories of how Medicaid has positively impacted the health and health care needs of Appalachians, the information from the site asserts that expanding Medicaid will provide all individuals in Appalachia the opportunity to live a healthy life with access to affordable, quality health care. Links are provided to the included states’ healthcare advocacy websites, with an invitation to readers to “get involved.” If supportive evidence for the need for Medicaid healthcare coverage access in Appalachia is needed, or interest in advocacy for this cause is desired, this site provides detailed information and compelling stories.

